# Digital transformation of home-based care: a narrative review of nursing, technology, and management synergies

**DOI:** 10.3389/fpubh.2026.1842444

**Published:** 2026-07-13

**Authors:** Fangfei Wang, Ronghui Zhu, Xin Sui, Jianying Jiang, Tingting Zhang, Zhenzhen Xu, Gohar Iqbal

**Affiliations:** 1 Shenzhen Nanshan Medical Group Headquarters, Shenzhen, Guangdong, China; 2 College of Social Sciences, Shenzhen University, Shenzhen, Guangdong, China; 3 Clinical Medical Academy, Changchun Medical College, Changchun, China; 4 Academy of Nursing, Changchun Medical College, Changchun, China; 5 Shenzhen Nanshan People’s Hospital, Shenzhen, China; 6 University of Veterinary and Animal Sciences, Lahore, Pakistan

**Keywords:** data-driven, digital transformation, digitally empowered nursing leadership, digitally proficient, interdisciplinary collaboration, technology

## Abstract

**Background:**

The transition from the home-based care model represents an unprecedented revolution in the healthcare industry, with the conventional clinical-based healthcare system being replaced by innovations in digital health. Yet there is no comprehensive analysis that explains how nursing practices, healthcare management, and the digital health domain work together along with the community-based care system. The data in this regard is fragmented. This review summarizes findings on integrating nursing, technology, and management to support chronic care.

**Methodology:**

A literature was screened by searching in PubMed, Scopus, and Web of Science databases using keywords such as “home-based primary care,” “nurse-led models,” “telehealth,” and “digital health equity,” with a focus on studies published between 2010 and 2025. The literature was searched for high-quality, peer-reviewed publications in English. The researchers selected 115 papers to conduct their in-depth study based on titles, abstracts, and full text.

**Results:**

These studies indicate that the shift from conventional home care methods to digital home care delivery requires nurses to develop digital skills to lead their teams while delivering personalized telehealth services. Nurses play a central role in coordinating care, facilitating patient engagement with effective technological developments that now provide better access to safer systems that enhance quality of life, while management frameworks support execution through policy development and resource allocation.

**Conclusion:**

The existing challenges include staff shortages, digital access problems, and obstacles created by existing regulations. Gaps still exist in the long-term cost-effectiveness of digital health, accessibility of resources equally to all areas, and effect of remote care on the relationship of nurses and patients. The field of home care requires resilient and equitable systems that need both predictive analytics and social health factor solutions and ongoing partnerships between different sectors. Home care will depend on nursing leaders with digital skills to ensure that technology helps them provide empathetic patient care.

## 1 Introduction

Healthcare is setting new trends. Delivery of healthcare services is no longer restricted to hospitals but also occurs at patients’ homes due to opportunities brought by digital health. An enormous number of services are being provided to patients at their doorstep rather than at hospitals. As a result, community-based care prioritized the patients. Community-based care has been established due to technology cherishing the interdisciplinary health services, personal agency, and long-term therapeutic relationships (1–7). Digital health framework is crucial for addressing the issues related to the necessities of older patients and those suffering from chronic conditions that cannot be fulfilled by hospitals (2–4, 6, 8, 9). The Backbone of community-based services is the home-based services that allow the individual to return to community-based self-care. To comprehend the domiciliary care with the passage of time, pillars are the provision of advanced nursing healthcare, rehabilitation, and compassionate supportive care at home.

For example, chronic conditions such as heart failures, breathing disorders and age-related illnesses can be managed if home care is administered in an organized manner and thus can improve the mobility and quality of life (2, 3, 10, 11). Patients are focusing on and enhancing home-based care as they can treat themselves at home (9, 11–13). Community-based care is divided into two major divisions: qualified nurses and robust management. The role of nurses is to examine the patients, develop care manuals and guide patients (2–4, 6, 14–17). Different studies indicate that nurse-led care leads to adherence to treatment, better outcomes and contentment (2–4, 16–20). Good management depends upon correct use of resources, evidence-based care, and coordination among teams (21–23). With the future of healthcare being prevention- and value-driven, effective management and application of new technology only get more imperative (6, 9, 22–30). Community-based care has altered due to amendments in the role of nursing management and technological developments in digital management. Digital health wearables, virtual consultation, medical mobile apps and distant patient monitoring increase the access, efficiency, and coordination in community healthcare, particularly with those suffering from chronic conditions and in remote areas (4, 6, 9, 29). Drawbacks such as digital knowledge, data confidentiality, and the need for continuous training of nurses and technological reliability still hinder (4, 24, 25, 29).

The application of co-creation strategies by the leaders would be required for initiating these technologies, as witnessed in the case of responding to the COVID-19 pandemic, as well as long-term care (24, 26).

Even with the rapid expansion of home-based care, plus the growing integration of digital health technologies, the literature still remains dispersed across domains, mostly across nursing practice, healthcare management, and digital health domains (1, 6). A lot of studies talk about these pieces separately, like telemedicine on one hand, chronic disease management on another, or nurse-led interventions somewhere else, but they do not quite explain how those parts run together inside community-based care systems (18, 27, 28, 31). Hence, there is fragmentation of existing data, and that has significant effects. Studying them separately limits our evidence about how home care systems perform. Telehealth interventions may show we can do something. But whether we have the nursing leadership, financial resources, and policy support to pull it off are factors typically not addressed within studies confined to one domain (6, 27, 29). Robust outcomes relevant to chronic disease management and reduction in hospitalization have been shown by nurse-led coordination models. Their scalability and durability heavily depend on the management guidelines and digital setup that are rarely observed together (14, 18, 20). There is a lack of integrative proofs regarding the design, grading, and funding of an effective home-based care system due to a communication gap between clinicians and officials and managers (1, 28).

So, consolidated synthesis is needed here to pull together evidence from these closely related areas and to make the combined function more obvious in relation to modern domiciliary healthcare. This review tries to close that gap by bringing together findings about nursing roles, management approaches, and digital health tools in home-based care, especially when we are thinking about chronic and older populations. It also aims to offer a more legible conceptual view of how these elements interact in practice to support better accessibility, continuity, and care quality within today’s healthcare systems. Although it has been established that digital and home-based care is beneficial for health, researchers still need to determine its effectiveness, efficiency, and equity across different life settings (25).

**Figure 1:**
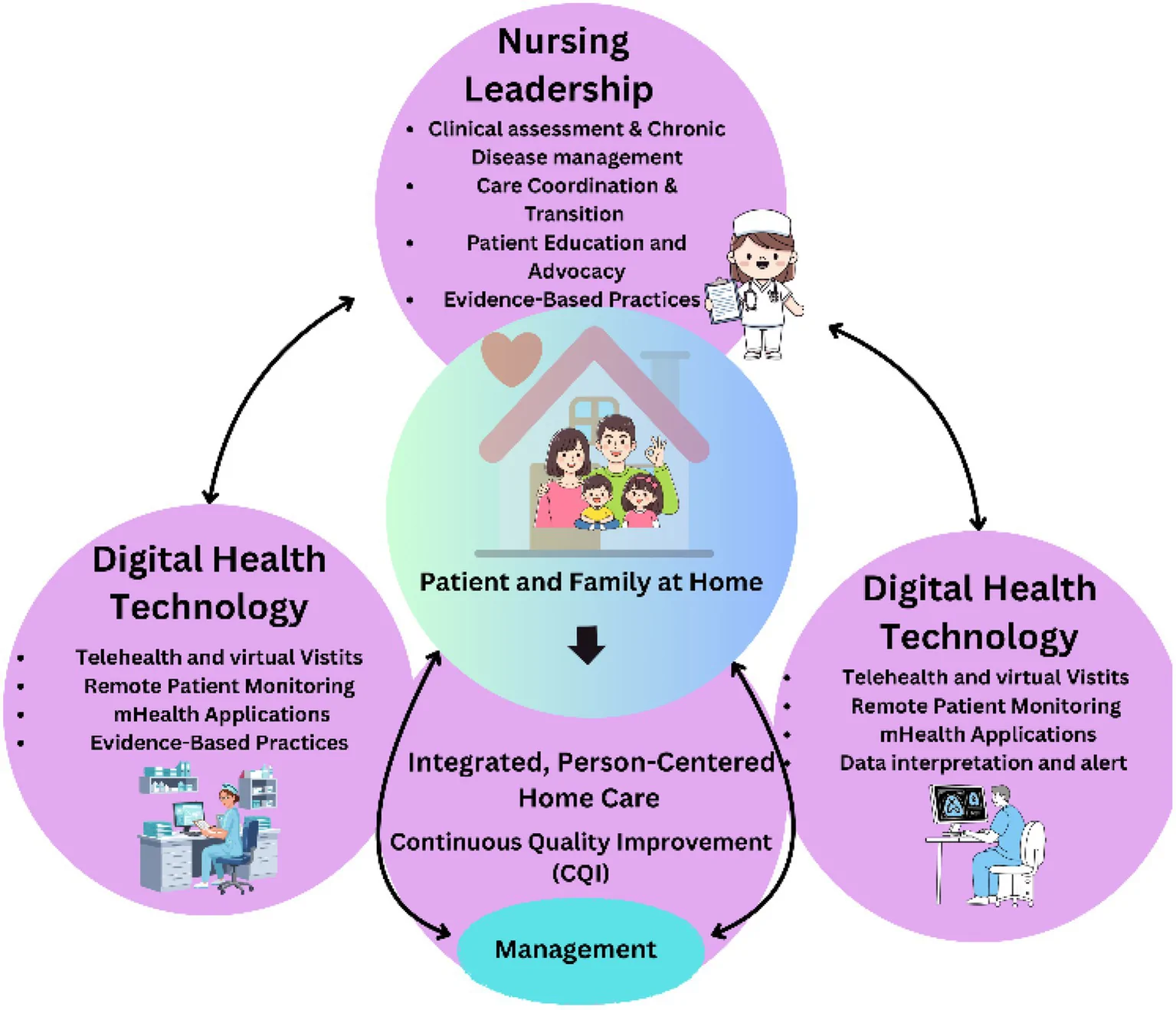
Presents the relationship between nursing, management, and technology and their ability to connect patients and families and ensure fairly sustainable care.

Cumulating together, the rationale for this review is threefold. Firstly, although research in this area is rapidly growing, it remains scattered across three domains: nursing, health informatics, and healthcare management, with little integration of their shared perceptions within home-based care (1, 6, 30). Secondly, older adults with chronic diseases are the populations that are contingent on home-based care and encounter multiple susceptibilities that require an integrated, multi-domain outline for effective intervention (2, 3, 8, 32, 33). Thirdly, rapidly growing digital technologies, such as wearable devices for monitoring health, use of AI, and extension of informatics, are important. So, nursing roles and management need to evolve as well, but how these are affecting their role and what is hindering it is still unclear (4, 27, 34–36). Through the concurrent addressing of these three gaps and mapping the functional relationship between management, nursing practices, and informatics, this review offers an engrossment that is more than a briefing of individual interventions. It acts as a comprehensible system of care delivery (Figure 1).

Table 1 in this matter provides a summary of the most significant advancements that portray the collaboration of nursing, management, and technology to redefine in-home healthcare services.

**Table 1:** Summary of key advances and their impact on community-based care.

Domain	Recent advances	Impact on patient outcomes	Key representative study	Study design	Supporting evidence
Nursing innovation	Expansion of advanced practice nursing roles; integration of evidence-based practice and digital health competencies	Improved care continuity; reduced readmissions; higher patient gratification	Abotaleb et al. (57) critical analysis of innovative nursing roles, practice strategies, and patient-centered attention	Critical analysis	(8, 14, 27, 28, 55, 57, 58, 61)
Management strategies	Programs for leadership development, efficient resource allocation, and coordinated team-based care.	Enhanced care coordination; improved satisfaction; higher quality care	Bhatti et al. (65) studied the influence of leadership style on staff satisfaction and inspiration in team-based primary care.	Observational study	(15, 16, 23, 26, 49, 53, 65, 66)
Technological integration	Telehealth platforms, mHealth applications, wearable devices, and electronic care plans	Expanded access and engagement, improved safety, and implementation of preventive measures.	Coffey et al. (20) developed and executed a nurse-led patient monitoring program remotely for ambulatory disease management.	Implementation study	(5, 20, 27, 29, 31, 35, 46, 87, 99)
Policy and regulation	Telehealth reimbursement reforms; expanded nursing practice authority; home-care accreditation standards	Greater equity, broader access, and more standardized care models.	Johnson et al. (28) evaluated nurse-led models of care within Medicaid to focus on enhancing the whole-person health and health equity	Policy analysis	(27, 28, 47, 76, 82, 90, 97, 109, 112, 113)
Education and training	Competency-focused curricula combined with simulation-based training and leadership development programs.	Enhanced workforce readiness; improved digital care delivery; stronger leadership	Melnyk et al. (56) developed evidence-based practice capabilities for licensed and advanced practice nurses in clinical setting	Competency framework study	(27, 36, 37, 54, 56, 62, 63, 66, 80, 114)
Integrated care models	Inter-professional teams combining nursing, social services, and primary care; case management pathways	Less fragmented care, better quality of life, and improved outcomes for patients and caregivers.	Deschodt et al. (18) establish core components and influence of nurse-led cohesive care models for older people with chronic disease by systematic review and meta-analysis	Systematic review and meta-analysis	(3, 14, 18, 39, 52, 59, 70, 71, 101)
Quality measurement	Value-based metrics, patient-reported outcomes, and readmission tracking	Data-driven improvements, strengthened accountability, and focused interventions.	Shetty et al. (76) observe quality enhancement activities and their association with performance outcomes by home health agency adoption	Cross-sectional study	(10, 21, 22, 49, 76, 77, 79, 103, 104)

## 2 Methodology

This study follows a narrative review approach to synthesize existing literature on home-based healthcare. A structured search strategy was used to identify relevant studies from PubMed, Scopus, and Web of Science databases. We used combinations of keywords such as “digital health transformation,” “home-based primary care,” “nurse-led models of care,” “telehealth,” “telemedicine,” “digital nursing leadership,” “remote patient monitoring,” “wearable health technologies,” “digital health equity,” “predictive analytics in healthcare,” “chronic disease management,” “nursing collaboration,” and “digital literacy and competency in nursing.” We did not consider articles that had a limited scope exclusively to hospital-based care settings, studies with insufficient methodological detail, or non-peer-reviewed. The latest view on the field of innovation and difficulties in community-based care from 2010 to 2025, peer-reviewed articles published in English, focusing on the role of nursing, technological solutions, and management in the home care setting, was considered. The literature was screened based on title and abstract relevance, followed by full-text evaluation to identify studies most aligned with the objectives of the review. In total, a broad body of literature was consulted, and 115 studies were ultimately included based on their relevance to the review’s thematic scope.

A transparent and planned search strategy was employed to ensure extensive coverage of the existing literature to map, join, and discuss the pattern available in literature rather than formally and statistically considering each study. Thus, interpretations of results are followed by narrative and thematic bases, and for this purpose, a PRISMA-style organized approach was used for screening to ensure transparency with database assortment to ensure reproducibility and consistency. Thematic relevance was part of the final selection of studies rather than a formal quality assessment checklist.

The selected studies were analyzed using a thematic narrative synthesis approach, looking for patterns in care delivery, technology integration, nursing roles, and organizational hurdles in home-based healthcare. Conclusions from included studies were assembled and discussed under thematic headings such as nursing innovations, management strategies, digital health tools, integrated care, quality of life, regulatory environment, and challenges construed by linking to one another to build an articulate, cross-domain narrative. In studies that showed similar findings, their patterns are highlighted. While studies show paradoxes in the literature are accredited and discussed rather than fixed through statistical weighting. This approach shows that home-based care is not simple; it includes complexity and multidiscipline. Thus, no single study domain can fully cover all aspects.

## 3 Overview of community-based home care

Home care is defined as the provision of health services to individuals by taking them to their homes. It includes medical and support services provided by health practitioners, such as nurses and therapists. It is possible to design a system that includes consideration of individuals’ health by integrating health services when leaving the hospital (8, 37, 38). Its main goals are to improve access, provide lifelong care, and improve the quality of life for people living with chronic conditions. It is also more cost-effective for the healthcare system (5, 39, 40). Stress and traveling will be avoided through home care. Complications and hospital readmission will be prevented through close monitoring. The studies prove that it reduces hospitalization and minimizes costs (10, 38, 41). Stokes et al. (10) confirmed through meta-analysis that case management for at-risk primary care patients significantly reduces hospital utilization, while Roberts et al. (39) found through an umbrella review that home-based delivery models constantly overtake hospital-centered approaches, though implementation fidelity remains a critical and often unaddressed regulating factor.

Home care services have developed multiple service models that meet different customer requirements. The services include nurse-led health services and personal care assistance and specialized palliative care and home rehabilitation services (4, 19, 40, 42). Personal care assistance helps individuals with their daily activities because it assists them throughout their entire day. Domiciliary care services provide chronic illness treatment to older patients through their home-based geriatric services. The older population uses personal care to maintain their ability to live independently. Palliative and hospice services provide advanced illness patients with both comfort and dignified treatment. Coordinated teams deliver their services at maximum effectiveness (12, 40). Home therapy could boost motivation and adherence in carrying out the rehabilitation plan (20). Dionne-Odom et al. (43) revealed through a randomized trial that telehealth-delivered reassuring care interventions stretched measurable benefit beyond the patient to family caretakers, an aspect that most home care models still imperfectly account for.

The community-based care system has expanded through technological advancements. People now widely use telehealth and digital monitoring systems and smart-home applications. Nurse-led telehealth enables nurses to perform vital sign assessments and provide counseling and immediate issue resolution (5, 6, 44, 45). This is especially beneficial for areas that lack sufficient services to cover their basic needs. The combination of virtual visits with self-reporting methods has emerged as a standard approach for chronic disease management, which helps improve diabetes and heart failure treatment results (40, 46, 47). Concerns have been on the rise when it comes to behavioral health and the mental health of patients is now viewed with the seriousness that is on par with physical health (32, 44, 48). Kennedy et al. (6) and Barnoy et al. (4) both noted, however, that nurses’ preparation for these technologies remains inconstant across settings, representing a tenacious gap between technological availability and frontline willingness that the literature has yet to fully resolve.

The emergence of home care was initiated by the need to decentralize the facilities provided in hospitals. Later on, the hospitals had to contend with a graying population with longevity and an increased incidence of chronic illness in the late 20th century. In the U. S., the policy reforms included the introduction of Medicare and Medicaid for the reimbursement of care outside the hospitals (1, 28). Discovering the relationship sustained in preventive care and team-based care postulated a specific ideal toward managed care and valued model during the decades of the 1990s and 2000s (28, 49). Digital transformation and population health improvements are driving the development of community-based models, which currently serve as the main framework for delivering equitable healthcare services with a focus on achieving positive patient outcomes (4, 33, 50).

Nurses have never stopped being essential in community-based digital empowerment. They provide clinical care, leadership, coordination, and education. Their flexibility spans clinical, behavioral, and social health, facilitating continuity and health literacy (14, 40, 51). Nurses achieve community engagement through their outreach work and health campaigns which also narrow gaps in social equity. Their experience and quality improvement activities make practice up to date (27, 52) aiding patient outcomes. Prajankett and Markaki (51) confirmed that advanced practice nursing is dominant to integrated older people care, with nurses functioning as system navigators across clinical and social dimensions, yet gaps in employees capacity and digital preparation continue to bound this potential in several community surroundings (53, 54).

## 4 Nursing innovations in community-based care

The way in which the community-based care is being redesigned fundamentally by the wave of nursing innovations is bringing to the forefront the expanded roles, evidence-based practice, and learning. The first ones in the list of APRNs, which include nurse practitioners (nurses who have acquired advanced education and can treat and diagnose health conditions) and clinical nurse specialists, are being recognized as individuals who can provide care to the patient with complex health conditions by themselves (44, 55, 56). These highly skilled experts diagnose medical conditions and recommend medications and develop treatment plans and deliver their specialized services to local neighborhoods (40, 44, 57, 58). Research also shows that the care provided by nurse practitioners reduces the number of repeated visits to the emergency room while involving patients and enhancing continuity of care. The same applies to clinical nurse specialists (nurses who have an advanced level of expertise in a particular area of practice and who are responsible for spearheading improvements in the delivery of care through the application of research findings in the following ways: through the application of principles of best practice, mentoring, and the assessment of patient outcomes in specialized fields such as gerontology and palliative care) (19, 40). Abotaleb et al. (57) and Clarke et al. (58) both confirmed that advanced nursing roles expand patient-centered outcomes, though they equally labeled that inconsistent scope-of-practice guidelines across regions remain a crucial barrier to identify the full potential of these roles at scale.

Beyond high-level positions, nurse-led care coordination models have also been proven to be disruptive. These models move the model of medical practice into a more patient-centered model in order to improve the communication between the health providers and the patients in the hospital (14, 59, 60). Nurses who actively monitor and treat patients with chronic diseases show improved patient results. The specific level of coordination needed to protect vulnerable groups, particularly older people who require ongoing care, needs continuous monitoring and collaborative efforts to maintain their well-being (52). Putra and Sandhi (14) found through a scoping review that nursing case management profoundly improves community access to care, yet noted that sustainability depends deeply on essential support structures that are commonly absent in under-resourced settings.

Evidence-based practice supports innovation and is a clear way to deliver nursing care that is both scientific and individual. In order to drive their elections, nurses are proficient in integrating research, clinical experience, and patient preferences (15, 27, 56, 61). EBP programs’ results showed better chronic illnesses management, enhanced reliability of care pathway and increased security in delivery of care (20, 40, 62). It was all due to skills such as a distant monitoring platform that provided nurses with immediate facts that allowed them to recognize the early signs and symptoms and alter their treatment roadmaps (6, 27). Ylimaki et al. (61) confirmed through a systematic review that advanced practice nurses show computable evidence-based capability, but recognized a tenacious gap between competence acquisition and reliable application in real-world community sites, a limitation that structured mentoring and continuing education have only partly talked (62).

Constant knowledge and development of leaders determine revolution as well. Advanced knowledge such as information sciences and telehealth, enhanced the openness and confidence and allowed them to deliver quality care by helping them (4, 6, 27, 63). Systematized mentoring and advanced information train nurses to work in teams and impact the health policies and make their presence in emergency settings more valuable than the bedside while using digital technology competently (27, 28). Enhanced quality care, increased retention rates of nurses, and higher leadership flow are linked with expert education in home health or management (49, 64).

Isidori et al. (63) found through a scoping review that while digital health competencies among nurses improved ominously during the COVID-19 pandemic, the gains were irregular across locations and largely informal, pointing to a breach in structured, curriculum-based digital training that remains unsettled in most community nursing programs.

Advanced roles, evidence-based care, new machineries, and continuous learning, which are included in nursing care, are changing home-based care into a more maintainable and efficient one. Nurses can improve patient outcomes, allow people to be in control of their health, and make health systems more effective, as clearly shown by the demonstrative programs led by nurses (40, 44). Nurses can contribute to the innovations of an equitable, contemporary civic health system and clearly indicate the increased roles of nurses in the near future.

## 5 Managerial strategies for effective home care

Excellent management of home care helps report the patients and direct the caregivers toward high-quality care. Workshops and awareness campaigns must be organized by the trained staff who guide the people about the hurdles associated with caring for someone at home (15, 62, 65). These leaders possess the integration of both clinical and strategic planning. Studies indicate that, through open communication, clear vision, and evidence-based decisions, teams feel satisfied, offer stable care, and minimize staff turnover (65, 66). To make employees comprehend how routine activities can be associated with something great and give them the wisdom of persistence is the primary mission. By resolving issues quickly, stronger trust can be formed through improved communication; this approach encourages safety and encourages innovation (52, 65). The best approach for making decisions is clinical evidence, frontline input, and information in real-time (66, 67). Another thing that is important is to empower your staff by educating them, defining career progression and rewarding them. Research also shows that mentoring and training leaders have a direct positive effect on nursing staff retention and on developing future leaders (14, 68, 69). Bhatti et al. (65) confirmed that leadership style directly figures staff satisfaction and motivation in team-based initial care, while Gusoff et al. (68) recognized job quality and organizational support as the strongest prognosticators of preservation in home care settings, both studies noticed that these factors remain contradictorily addressed across agencies, representing a tenacious management gap.

The community-based care system requires a multidisciplinary team-based approach to ensure comprehensive patient care because their medical, psychological, and social needs must be addressed (52, 70, 71). Well-articulated roles and transparent decision-making procedures, which result in shared decision-making, reduce duplication and promote mutual respect. Patient satisfaction, improved treatment adherence, and reduced hospitalizations are often seen in models that involve regular team meetings and cooperative care planning (4, 33). In reality, the optimal resilient and creative home care teams are formulated by leaders who respect, empathize, and work toward inclusivity (38, 72). DePace et al. (72) found that communal leadership models in home-based soothing care specifically advance APRN roles and improve team unity, yet noted that such models depend on clearly defined governance structures that many community-based organizations have yet to start.

Intelligent operations and the distribution of resources constitute the foundational framework of sustainable care. This means that the leaders will need to align their staff, technology, and supplies with the needs of the patients, creating models of care in the process. The leaders will need to be focused on developing dynamic strategies that are sensitive to emerging trends and technology (41, 73). Calculated planning, like early exposure of staffing risk, can be used to avoid facility interruption to susceptible patients (10, 28, 74). A sustainable foundation in the long term and trust can be achieved through clear accountability (66). Digital tools enhance coordination and monitoring in home-based care (5, 27, 75). Together, these technologies create a continuous, data-driven care system rather than isolated interventions. For example, Access to tangible information fosters very quick realization of idea initiatives (20). Healthcare innovation will be an active coordinating tool, quality assurance device, and a patient protection device under virtuous management support (67). Continuous quality improvement is the prime driver of good management. Higher management should put in procedures for daily reviewing the quality of care, patient response and staff performance (62, 76, 77). Xia et al. (67) established through a machine learning approach that estimating home care quality across community health service centers is achievable and yields illegal insights, though the authors alerted that data standardization across agencies remains deficient to enable reliable system-wide benchmarking.

Measurement of determining factors that include infection rates and readmissions permits the enforcement of specific advances that can meet high standards (10, 40, 67). The innovative practices that organizations use to build a culture that combines responsibility with creativity enable their frontline staff to design effective solutions. The programs, which operate at full capacity, require input from patients and their families to improve both care coordination and family and patient satisfaction levels (12, 40, 78). Regular audits and accreditation reviews enhanced the quality results and practices (4).

Leaders who communicate effectively with their staff members while providing them support and development opportunities create organizational systems that will effectively manage all upcoming challenges (15, 62, 79). These people blend digital tools, research, and empathy to ensure that everyone receiving care from home can lead healthy, productive lives. Kitson et al. (15) found across a four-country study that nursing leaders who keenly promote evidence-based practice at point-of-care pointedly expand execution outcomes, although acknowledged that leadership volume for this role is unequally distributed and often underdeveloped in community sets, a gap that targeted leadership development programs must urgently discourse.

## 6 The digital health toolkit: telehealth, mHealth, and wearables

The technological revolution is transforming home-based care by delivering more personal and proactive and accessible services. The last decade has seen telehealth and mobile health applications and wearable devices develop into tools that provide continuous medical support instead of delivering on-demand treatment (4, 5, 35). Real-time communication with patients, providing distant assessment, and intervening immediately without being present with a patient is the result of these technological devices for nurses. The application of interlacing technological innovation in the daily road map has contributed to the realization of clinical indicators of patient satisfaction, commitment to treatment, and health consequences (27, 80). Robles-Aguilar et al. (80) found through a qualitative meta-synthesis that primary care nurses report both significant benefits and extensive burdens from digital health incorporation, with documentation demands frequently diminishing the relational dimensions of care a tension that the extensive literature has yet to effectively resolve.

Telecare has recognized itself as a cornerstone of a care delivery. It establishes critical channels of virtual consultation which enable professionals to monitor patient progress and provide educational materials to users. The evidence shows that the treatment improves chronic diseases while decreasing hospital readmissions for heart failure and diabetes patients (20, 35). Nurse-led programs are predominantly effective, as they permit complex assessment and patient education through video conferencing associated with electronic records (40, 44, 81). Such models provide remote areas with high-quality medical services which promote health equality. The system needs organizations to establish three essential components: infrastructure, privacy measures and complete training for all personnel to achieve its objectives (40, 54, 82). Ma et al. (40) confirmed through an integrative systematic review that nurse-delivered telehealth in home-based calming care is both effective and acceptable, while Wilson et al. (82) recognized through a systematic review of qualitative studies that progressing digital health fairness needs targeted structural reforms, including device access, digital literacy support, and culturally responsive design that most recent telehealth programs still lack.

In practice, the actual results demonstrate overwhelming evidence of success. Structured telemonitoring programs show two benefits because they enhance patient satisfaction while decreasing emergency room visits and developing self-management abilities (35, 41). In like manner, tele-integrated mental services enhance attendance and adherence to treatment (44). In the case of rural dwellers, online visits decrease the load on transportation and enhance the level of access to specialists (5, 41). Telehealth goes beyond a tool; currently, patient-centered care is fundamentally essential in community health (80). Mallow et al. (41) proved that community-based telehealth is practicable and acceptable for preventing long-term care readmission, though the authors warned that scalability beyond pilot settings remains an unresolved challenge requiring continued organizational and policy investment.

However, patient engagement has also undergone a significant shift in terms of mobile health, or mHealth, applications. Patients can record their symptoms, receive medication reminders, and learn using the applications provided by their smartphones (7, 35, 46). The applications provide vital assistance for people with chronic diseases because they enable patients to monitor their conditions and receive guidance to manage their health (7). This virtual environment is vital, as the nurse can make decisions on the choice of applications, data explanation, and revise care plans as necessary. These platforms offer safe communication channels and, hence, offer quick solutions to health concerns and minimize clinic visits, especially among the less mobile and in underserved areas (33, 35). One of the earliest papers on nurses’ attitudes about telehealth was conducted by Bashir and Bastola (7). In this pilot study, nurses shared neutral-to-positive perceptions about telehealth effectiveness but frequently questioned telehealth impacts on quality of care and workflows, issues that remain today even after years of technological development. Mobile Health (mHealth) enables educational programs that lead to permanent behavior modifications while also providing monitoring abilities.

Mobile Health (mHealth) enables educational programs that lead to permanent behavior modifications while also providing monitoring abilities. The diabetes applications enable users to track their glucose levels while receiving guidance on their daily activities. COPD applications specifically target user needs by monitoring their physical activities and medication intake. Health records that include patient-generated data empower healthcare professionals to make better evidence-based decisions through enhanced data analysis capabilities (27, 83). Guo et al. (84) has proven with an RCT that a smart and connected aging model can improve quality of life outcomes for older adults living at home. However, replications have only been done in high-resourced settings – leaving a lack of evidence if these findings can be applied to community settings with fewer resources.

These are innovative devices that monitor heart rates, blood pressure, oxygen levels, and even the quality of sleep and provide evidence between visits to the doctors (4, 5, 35). The combination of activity monitoring and fall detection technology helps older adults maintain better health while preserving their ability to live independently within systems that provide coordinated care (38, 85). In addition, wearables can assist in making patients more active, and this can be done through the use of immediate feedback on their activity or compliance level, which will encourage them to engage in more healthy activities. This can be maintained through the use of gamification with set goals and progress tracking (46). Nurses use this enriched data during consultations to provide personalized counseling and create tailored care plans. The integrated systems that send clinical alerts automatically enable continuous monitoring while reducing the need for in-person visits which costs fewer resources (20, 83). The emerging technology in the area of innovation is highly predictive in the form of artificial intelligence (AI) and machine learning. The technology has the ability to analyze record and wearable data to identify patterns, stratify risk, and be proactive in terms of intervention (86). The same is reconciled with the One Digital Health initiative that encompasses a holistic dataset integrating human, animal, and environmental data (87). Finco et al. (85) advised that even if wearable and monitoring technologies really do give clinical benefits for older adults, their use also brings uncertain ethical questions about consent and who owns the data and, then, surveillance too and current governance frameworks just have not addressed it enough. The emerging technology in the area of innovation is highly predictive in the form of artificial intelligence (AI) and machine learning.

Nonetheless, this digital revolution also has its human side effects. Certain applications, such as Electronic Health Records that are supposed to simplify nursing practice, could be a primary cause of nurse burnout because of their focus on documentation as opposed to spending time with patients (35, 80). The healthcare system needs to address its second challenge which exists as a digital divide. The current requirement for social inclusion now functions as a fundamental element that determines health outcomes. The existing health disparities will expand because the most vulnerable groups cannot access essential benefits due to their lack of broadband access, electronic skills and ability to use technology (82, 85, 88, 89). Yao et al. (89) recognized a measurable inequity in the access to health facilities based on the implementation of digital health technologies through a scoping review. Fox and Connolly (88) reported that alterations in generational mHealth adoption continue to exist and need age-sensitive implementation approaches, which most lack.

The implementation of technology in community-based care settings requires more than just the equipment itself, and it should be accompanied by a caring and patient approach as well. Technology should enhance, not replace, human interaction in care, maintaining empathy even in remote settings (80). A caring, state-of-the-art digital future in in-home healthcare services should strive for improvement of technology while still putting social justice and human relations in the forefront (35, 85).

Combining all these, the paradigm shift is formed by telehealth, mHealth, and wearables. The considerate combination of all these has brought remote monitoring and the prevention of chronic illnesses to a new height, never before seen in history. The sustainability of the implementation, however, requires continuous staff training, strategic investment, and strong policies on data privacy and cybersecurity (85, 90, 91). The future holds an understanding of interdisciplinary professional engagements so that technologies can work in concert with clinical processes while making sure that access is even. The combination of assistive technologies with skilled nursing professionals will remain essential for achieving sustainable health solutions that meet specific community requirements (4, 35, 40). Chaet et al. (90) and Abdul-Rahim and Alshahrani (91) both recognized that ethical and supervisory frameworks for telehealth remain segmented and applied across authorities contradictorily. This affects the safe and equitable scaling of home-based care at the system level, which is a functional undetermined foundational gap.

## 7 Integrated care approaches

The central stage of effective home-based care has now become integrated care, where high-quality medical, social, and rehabilitative care are combined in a cohesive plan. This approach is based on addressing the issue of fragmentation in care, such that the patient is provided with continuity and holistic care (52, 74, 92). The existence of strong evidence demonstrates that integrated care systems improve three outcomes, which include chronic disease management, patient satisfaction and prevention of unnecessary hospital admissions (1, 18, 74). Hughes et al. (1) established through a systematic hermeneutic review that shows combined care strategies have potential effects on healthcare that is heavily influenced by different contexts. Their effectiveness is reduced by issues at the managerial level and lack of proper implementation and lack of clear responsibility among different organizations.

The staff members work at home because they can monitor health conditions throughout the day which enables them to detect health changes that require immediate medical attention (8, 14, 18). Building patient confidence in self-management, urgent visits and compliance can be increased by nurses’ contribution (40, 93, 94). Apart from clinical outcomes, the knowledge and compassion of the nurses contribute to the emotional stability and success of the interaction with the patients.

The effective integration implies the actual existence of collaboration among the disciplines. There is no profession that will be able to serve all the patients. The research confirmed the effectiveness of the inclusive planning, where the nurses make use of the feedback from physiotherapists, dietitians, and social workers and perform more holistic and flexible interventions (5, 39, 60). Nurses use their expertise to transform advanced medical instructions into daily care routines while establishing effective communication pathways for doctors, patients, and their families. Integrated Care Pathways (ICPs) serve as research-backed structures that define responsibilities and create schedules and initiate standard operating procedures while maintaining respect for personal goals (20, 27, 49). When nurses are involved in the design and monitoring of these pathways, care would be more consistent, efficient, and responsive. This should be applied to patients and caregivers and should improve health literacy or shared accountability (28, 92). Slatsveen et al. (71) conclude that interdisciplinary frontline workers face the contradictions between organizational work and trust-based collaboration, indicating that interprofessional goodwill is as important as structure design of integrated teams through a qualitative study.

Continued assessment and technology are another area of strength in the concept of integrated care. Medical records and other technologies, such as the use of electronic health records and electronic dashboards, make it possible to share information in real time (5, 27, 95). Constant enhancement has a feedback loop of tracking outcomes like readmissions and gratification (18, 40, 96). The analysis process requires patient involvement because it produces transparent results and establishes shared responsibility between stakeholders. All this can never happen without committed management and leadership. Managers establish interdisciplinary coordination frameworks that include formal communication procedures, performance control systems and shared information networks. The ability to coordinate operations while reducing duplicate work improves through regular interdisciplinary meetings, joint training sessions and shared documentation (15, 49, 97). Those leaders who emphasize trust and inclusiveness and use evidence-based decisions will make decisions that match the needs of patients. Mobasseri et al. (96) demonstrated through a multi-method study, the model of home-based long-term care will not be effectively conceived unless the three aspects of technological, organizational, and policy are considered at the same time. It has shown that the model considering one aspect at a time is incapable of achieving sustainability, an inadequacy that many integrated care literature sources failed to address.

In brief, integrated care is a powerful integrative process that integrates nursing, management, and technology. Through collaboration and technology, and with a focus on meeting the goals of patients, it establishes a process that is continuous, equitable, and sustainable (38, 52, 74, 92). It is a fundamental redesign of the home care of health and well-being. Sarri et al. (74) established that the integration of home and community-based care has a positive impact on quadruple aim outcomes and equitable health across health system but still cautioned that equality is still not reach to the population who required it the most due to uneven distribution and current integrated care model.

## 8 Nursing-led management improves the quality of life

**Figure 2:**
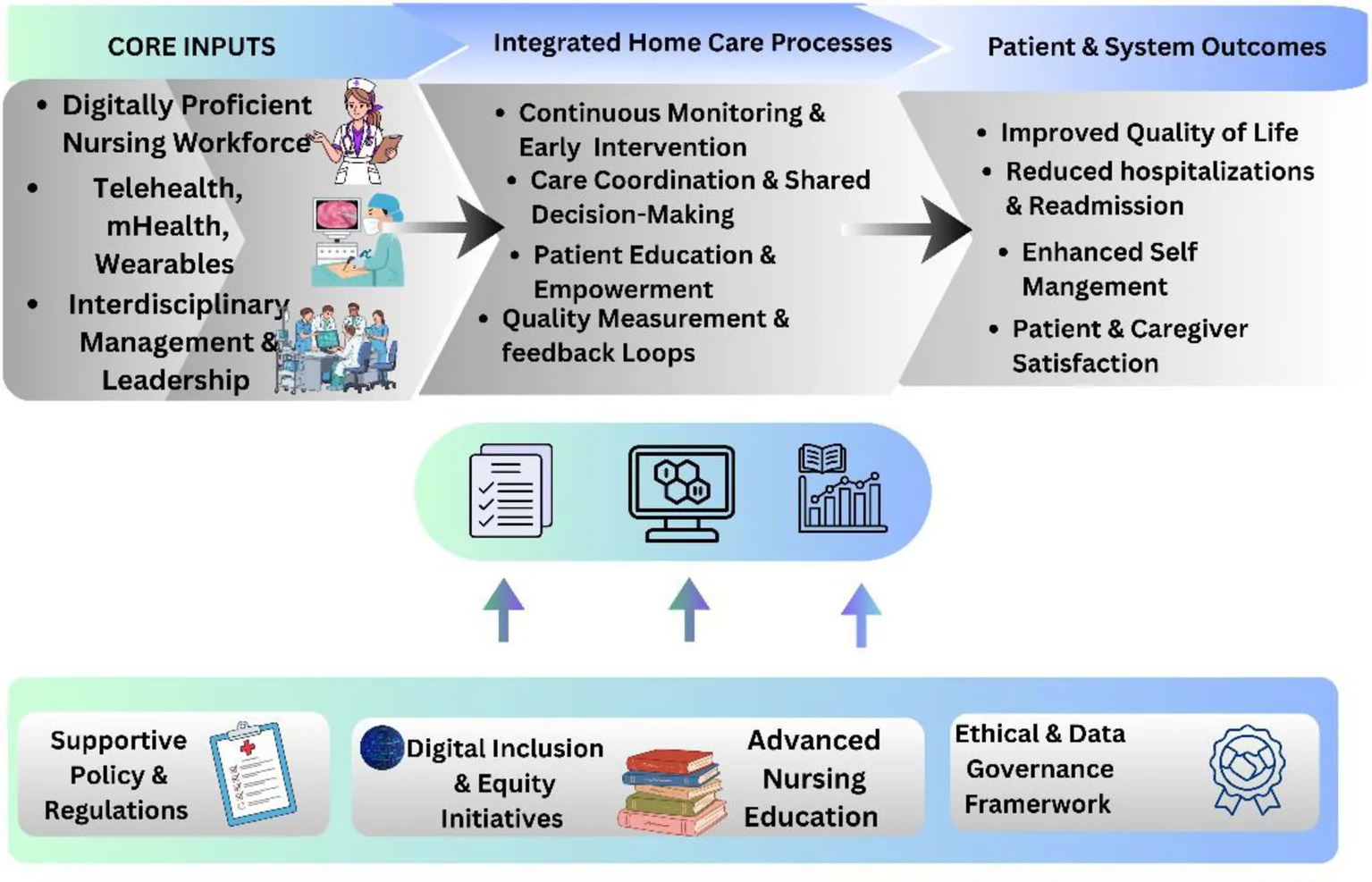
Illustrates the sequential pathways through which digitally enabled, nurse-led home care translates into improved patient and system outcomes.

The primary purpose of patient-centered home services is to help individuals live a quality life and manage their chronic disease (8, 14, 18, 94, 98). It is an emotional burden associated with illness in the long run, which in most cases results in depression, anxiety, and isolation. The role of nurses in providing a lifeline cannot be overstated because they are able to identify mental health challenges and connect people with vital support systems through routine screenings and pre-established routes of referral (12, 44). The research proves that properly structured psychosocial assistance, which allows remote health services to deliver support to patients, results in two benefits for patients and their family caregivers. The complete treatment approach enables the entire family to manage their difficult experience with the illness (40, 98–100). Wong et al. (100) concluded in their systematic review and meta-analysis that quality of life among community-dwelling older adults has significantly improved through nurse-led telehealth self-care promotion programs, while Abdel-Aziz et al. (98) identified that persistent gaps reduce the quality of life due to palliative care, particularly around psychosocial support and family caregivers in community-based settings given by nursing-based review (Figure 2).

Improvement of life occurs through full engagement. Teams of nurses who work with social workers and therapists are able to design care plans that meet the medical and social needs of patients (14, 18, 101, 102). These enduring developments require an investment in the labor force and an increase in care management to make them sustainable (62, 103). Practical interventions are the most active ones in some cases. Nurses usually test households for falls and other risks and refer families to the simplest changes that leave them independent and help to avoid accidents. The combination of these adaptations with regular follow-ups enables people to maintain safety without needing hospital treatment (18, 38, 101, 102). With such tools as remote monitoring being introduced, it is also possible to envision even greater improvements in terms of medication adherence and safety incidents that will result in a reduced level of stress for patients and families (20, 41, 99, 100). Pendet et al. (102) through a systematic hermeneutic synthesis, researchers discovered that while integrated care models appear promising, translation to community settings is somewhat dependent on context. Often, they have not shown great success due to implementation issues. A recurring issue is lack of sharing. Leadership/responsibility between organizations.

Nurses work to improve community health through their activities, which extend beyond hospital environments. The organization provides three different programs, which include workshops, support groups and resource system navigation guidance to its clients. The programs help people overcome loneliness while they learn to manage their health because health management acts as a key factor for living a healthier life (14, 32, 98). Nurse leaders will dedicate their maximum efforts to provide equitable service access while ensuring that healthcare services reach their most vulnerable patients and effectively meet community needs (28). However, it is not necessarily a guarantee of quality of life that benefits all individuals. Continuing disparities, especially regarding dementia care, are a reminder of the need for us to work hard at breaking up these barriers of access. Without working hard at addressing social determinants of health, they will continue to be left behind (18, 32). The development of nursing-designed models that combine outreach programs with equity initiatives delivers better results for reaching underrepresented populations while generating more extensive beneficial impacts (8, 28). Abdel-Aziz et al. (98) emphasized that addressing deficiencies in community-based supportive nursing, especially in managing social isolation, caregiver stress, and equal access to care and support services, is crucial for ensuring nurse-led community-based initiatives effectively enhance the quality of life of vulnerable populations, an issue that is inadequately addressed by existing models.

All in all, it can be concluded that emotional support, working as a team, practical home modifications, and involvement in the community have a positive effect on the quality of life of people who are provided with home-based care. Instead of focusing on just one of these, it is best to support dignity and independence through consistent integration and prioritization of patient-recognized needs (18, 94, 98, 100, 103). Rico-Blázquez et al. (94) established, by implementing the pragmatic cluster-randomized controlled trial in primary care, that the combination of home-based nursing support and cognitive restructuring results in better quality of life of family caregivers in primary care. This highlighted that in home-based care, outcomes of quality of life cannot be completely understood without clearly covering the opportunity of nursing intervention so that informal caregivers are considered.

## 9 Regulatory environment and policy

Home care regulations extend beyond their documentation because they establish the hidden framework that either assists or restricts nurses during their home care activities (90, 104). These policies are “must-have” safety nets for the healthcare system, given that it often cannot meet the varied requirements of the communities in a dynamic response to problems that would arise from care (28, 104). However, there is an encouraging change in progress, and the policy has become more focused on prevention, control of chronic diseases, and interest in helping everyone live long enough to grow old at home. This is a simple step in a new approach to care that seeks to abandon the traditional model of care in favor of one that takes account of the way people live their lives (28, 38, 104). The funding structure of care delivery, together with its implementation, will determine who ultimately receives necessary care solutions according to the existing policy. Haferkamp et al. (38) established that in 2025 home-based primary care has become a key component of aging-in-place policy; so far it is recognized that workforce policy and reimbursement models have not evolved with the clinical evidence, which leads to a structural gap research-supported practices and current policy implementation.

The U. S. Medicare Home Health Benefit establishes eligibility criteria for patients and determines how much healthcare providers will be paid, which directly affects daily operational procedures through its established policies (27, 104). It is not simply a bureaucratic reality; it is a determinant of how much money home health agencies will be financially feasible and how much nursing intervention will be sustainable. The Affordable Care Act has also increased access points through prioritizing preventive care and transforming nursing practice into a more autonomous care coordination approach and patient coaching approach (10, 14, 104). The state licensing system creates operational difficulties because it uses a complex network of regulations to restrict its functionality. The regulations establish professional competency standards through their implementation, but they create confusion for nurses, which prevents them from delivering uninterrupted telehealth services to patients across state lines, thus harming patient care continuity (27, 90, 104). Another group of nurses who bear the burden of the regulatory body is the frontline nurses. The massive documentation and compliance time for the frontline nurses constitutes non-patient time, which results in inefficiencies and the loss of skilled nurses from the community-based care occupation (27, 66, 69, 105). This effort will render community-based practice objectionable to the future generation of nurses. The regulations need reevaluation through better remote systems, which should be made easier to use in process management. Health record interoperability policies enable health professionals to reduce unnecessary paperwork while dedicating more time to direct patient care (20, 105). Chaet et al. (90) proved that ethical and regulatory clarity is foundational to maintainable digital health practice, while David-Olawade et al. (27) know that fragmented licensure and inconsistent, poor policies across authorities remain among the most important reported hurdles to scaling nurse-led telehealth in community settings. Policy reforms have limited data, and gaps exist.

The strategy should be developed in three key parts in order to create an improved system. First of all, we need to adopt technology. Although telecare is basically necessary, compensation contexts have not kept up, which restricts its potential (27, 104). In order to support virtual health, there should be equitable compensation for virtual visits, as well as investment in virtual literacy, based on the body of research that supports the policy-affirming electronic technologies can lead to reduced hospitalizations and improved care for people with chronic conditions (41, 104). Second, the future of the workforce relies on the plan to recruit and retain staff. Debt relief and rural incentives can help in reducing the shortage of labor (66, 106). Simple regulatory frameworks and strict mentoring system have also been effective in increasing the Less complex regulatory demands and strict mentoring systems have also proven to be effective in enhancing retention rates (56, 106). Lastly and most importantly, policies should be quality-oriented. A specific group such as aged people, low-income families, and the chronic disabilities category, still hinders the acquisition of proper health care distantly (32, 33, 104). Policymakers need to develop funding systems that distribute resources fairly, while they should establish outreach programs that need to provide cultural competence training to nursing students so that home-based services become accessible to all individuals (28, 106). Morris et al. (66) via rapid review, identified that workforce recruitment and retention into professional care careers are severely lacking support from current policy environments. Supervisory overload and lack of mentorship opportunities have been identified as the most actionable drivers (and thus far least attended to) of attrition from community nursing.

Finally, we have the policy and regulatory environment, which is a significant force that enables or hinders the growth of nursing under community-based home care. Through strategic regulation and innovation in areas such as telehealth, human resource development, and patient-centered equity, we are able to build sustainable systems that empower nurses and patients and provide quality and safety in the delivery of care at home (27, 58, 104, 105). It will all come down to harmonizing between certain responsibilities and adequate pliability in the future. Clarke et al. (58) reasoned that contemporary nurse-led models of care need regulatory environments that clearly recognize and support extended nursing autonomy, and that without such acknowledgment, the potential of nursing to enterprise equitable, sustainable home-based care will continue to be controlled by frameworks intended for a different era of healthcare delivery.

## 10 Challenges and barriers in nursing and management

The community-based home care facility faces serious obstacles that challenge its staff members and their work with at-risk populations, despite the advancements that have been achieved throughout history. The two obstacles exist because of their relationship with two factors, which include unfair access to critical medical services and the second factor, which involves unpredictable financial support that creates a domino effect, which harms patient results, staff health, and system strength (32, 33, 107). The most obvious issue of access to care is highly discriminatory. There is a delay in diagnosing patients in rural areas and deprived areas of cities because patients have to travel long distances, and specialized nursing care is very rare; therefore, patients lack good health results (107, 108). The nurse shortage crisis creates more difficulties for caregivers because it creates more barriers that prevent people from accessing medical services. The frontline staff members frequently have to manage relatively huge loads of cases with minimal backup because this combination creates a situation that will ultimately lead to their exhaustion and result in diminished quality of their work (66, 69, 107). Cabañero-Garcia et al. (107) confirmed through a systematic review of reviews that fences to health, social, and long-term care admittance among older adults are multi-layered and equally reinforcing and that geographic, financial, and workforce-related barriers are gathering unreasonably in the same communities, compounding disadvantage in ways that single-domain intrusions consistently fail to address. Gebhard and Herz (69) further found through a two-decade systematic review that the health of home care workers themselves remains frequently under-prioritized in policy and research, representing a gap with direct values for workforce sustainability and care quality.

These barriers are not limited by geography. With respect to poor families and older people, the issue of transport, lack of digital skills, and insurance becomes a financial burden on the continued use of community-based care. Patient isolation can also arise as a result of cultural and language barriers, which create communication barriers and limit full participation in patient care (33, 107). Mobile clinics and community-based care showed promising solutions that can close these gaps and offer a lifeline to underprivileged populations (41, 109). The work now presents greater complexity and increased difficulty because its current requirements exceed available capacity. The virtual technology solutions that were developed to provide assistance have created additional challenges for users. Nurses currently face two main challenges. Their first challenge involves managing multiple platforms which requires them to constantly monitor their work. Their second challenge stems from the need to handle excessive documentation that comes with electronic systems which creates a major risk of exhaustion (110, 111). This stress is deteriorated by a perception of a gap in preparation; the nurses perceived they are not equipped to deal with the multifaceted situations they might encounter at home, to treat multiple chronic conditions, and to offer palliative care through telecare (7, 27). The traditional nursing practices may be a failure in preparing the graduates for the certainties of community practice, and digital-savviness is now a necessity (15, 56, 109). The combination of increased nurse workload and continuous administrative duties creates conditions that lead to nurse burnout and absenteeism which directly affect the quality of patient care (66, 109). The crisis is therefore a result of a lack of professional development support or good management support. It has also been observed that having a top-notch mentorship program and continuing education is not a luxury but a necessity for retaining nurses in the profession and ensuring patients receive parallel results (56, 109). Harris et al. (110) and Abraham et al. (111) concluded that EHR-induced stress was a significant and independent predictor of burnout in advanced practice nurses and primary care nurses. Abraham et al. further showed that extensive use of EHR systems enhance the hazard of burnout among nurse practitioners. This result highlights a problem in the design and implementation of these technologies that have not adequately addressed by healthcare organizations and technology developers.

The financial threat of impending insecurity serves as the hidden danger that exists behind everything. Most home-care agencies operate their businesses through financial fundamental uncertainty which depends on the changing patterns of Medicare and Medicaid and private insurance companies (28). The agencies have to condense the labor force, services, and postpone vital stashes in training and technology when compensation becomes smaller or decreases (66). Low-income agencies with minimal employees are directly linked with a higher proportion of dissatisfied patients and enhanced reentry to the hospitals (10). Lastly, there is the risk that the digital insurgency will leave the most susceptible behind. Writing digital access, the ability to connect to something, and the tools and knowledge to do so, has become a basis of health (86, 107, 112, 113). The digital divide creates additional social disparities because urban locations receive total financial support while rural areas and low-income communities remain dependent on outdated technologies. The combination of data-driven resource distribution and new partnership approaches will create solutions that deliver technological advantages to all people (33, 86, 109). Sieck et al. (112) established that digital technologies are an important factor in health settings that directly affect the well-being and health of people, while Lyles et al. (113) another study, give the similar finding in JAMA that proper implementation and equality of digital health require robust policy and facilities. In short, both studies conclude that digital health can improve the outcomes and does not risk the investment until equitable access can be achieved.

These combined problems require an intensive effort. A long-lasting coordination between policy makers, medical organizations, and professional associations will be essential. It will establish a secure subsidy base, justify access and well-supported personnel. This approach is obvious, and nurse leadership, together with strategic funding, should serve as the obligatory requirements that communities require for delivering high-quality patient-centered healthcare services (28, 66, 86, 107). Table 2 takes these demanding issues and yields evidence-based, effective avenues, producing a comprehensive and easy-to-track agenda for constructing a better tomorrow. Gallegos-Rejas et al. (109) synthesized evidence from interdisciplinary perspectives that inequality of informatics can be reduced by communication between clinicians, policymakers, and technology innovators and communities as well. This suggests that these challenges affect the entire healthcare system and require coordinated solutions rather than individual or discipline-specific approaches.

**Table 2:** Ongoing challenges and pathways forward in community-based care.

Challenge area	Current status	Recommended action	Key representative study	Key finding	Supporting evidence
Access disparities	Rural and low-income communities have restricted access to community nursing services and reliable broadband.	Expand telehealth infrastructure; implement mobile clinics; develop equity models	Gallegos-Rejas et al. (109)	A multidisciplinary coordination approach is important to reduce the digital division and establish the equal access to digital resources across all societies.	(32, 33, 86, 88, 89, 107–109, 112, 113)
Personnel shortage	Nursing shortages, burnout, and aging workforce threaten care sustainability	Implement mentorship programs; create career ladders; flexible scheduling	Morris et al. (66)	Workforce enrollment and retention can improve the evidence based professional care by different policies such as flexible scheduling, Mentorship programs, and organized education	(48, 53, 56, 66, 68, 69, 78, 106, 110, 111)
Funding constraints	Education of staff and adoption of new technologies can be interrupted due to limited funding.	Develop public-private partnerships; outcome-based reimbursement models	Lizano-Díez et al. (13)	Measurable positive effects are observed on patient outcomes and economic indicators that further strengthen the evidence for sustained investment by home care services	(10, 13, 28, 66, 74, 76, 92, 96)
Policy gaps	Fragmented licensure and telehealth policies across regions	Establish standardized telehealth frameworks and use best practices as benchmarks.	Chaet et al. (90)	Ethical and regulatory precision in mhealth practice is initial to building structure, trustworthy cross-regional care outlines	(27, 28, 44, 46, 47, 90, 97)
Technology hurdles	Resistance to change, inadequate training, and cybersecurity threats	Implementation science studies; secure intuitive systems	Krick et al. (25)	Training quality and implementation support directly influence the acceptance, effectiveness, competency of digital care technologies	(7, 9, 24, 25, 27, 80, 85, 91)
Care coordination	Poor coordination and communication among hospitals, primary care providers, and community teams.	Develop interoperable systems; collaborative protocols	Hughes et al. (1)	To efficiently reduced the segmentation across care setting required the integrated care approached that includes system level thinking and context-sensitive implementation	(1, 18, 20, 52, 70, 71, 74, 102, 115)
Outcome measurement	Inconsistent metrics make effective comparison and benchmarking difficult.	Establish core outcome sets for research	Shetty et al. (76)	Measurable recital improvements were observed by the home health agencies that implement the quality improvements activities and improve patient outcomes as indicators	(10, 15, 49, 67, 76, 77, 79, 93, 103)
Ethical concerns	Privacy, data ownership, and consent issues in home monitoring	Develop ethical frameworks; assess consent models	Finco et al. (85)	Privacy, autonomy, and concerns are the challenges that arise during older adult care by digital technology and require the proactive framework.	(27, 37, 85, 87, 90, 91, 114)
Cultural barriers	Models lack cultural adaptation; insufficient caregiver support	Collaboratively develop culturally sensitive care models while reducing the burden on caregivers.	Giebel et al. (32)	Community-based care is not equally accessible, particularly for dementia patients, due to culture and structure barriers that remain unaddressed	(28, 32, 33, 42, 55, 69, 94, 107)

## 11 Discussion, future directions, and research opportunities

This review predicts the consistent and theoretically significant pattern evidence based on the fact that nursing practices, healthcare management, and digital informatics are not independent fields; they are linked with each other in a single structure system. When these interdependent domains are followed and actively managed in home-based care then data clearly shows the better chronic disease management, improved access to facilities, enhanced quality of life and patient satisfaction (49, 61, 66, 109). When these domains are not actively managed such as digital technology is implemented without proper nursing training and their duties are increase without managerial support then literature shows that constantly limited outcomes, unsuitable results and unequal distribution of resources among patients (1, 6, 36, 107). It is the integration of systems point of view that forms the main theoretical contribution of this review paper. Instead of viewing the problem of home-based care improvement in terms of enhancing one innovation and one profession, this problem is viewed through the lens of the development of all three systems at once, something that previous research literature has failed to address properly (28, 56). The field of home care development creates fresh opportunities for healthcare design, which demands new approaches from both nursing and administrative fields. Our future needs to establish evidence-based systems that provide equitable solutions that people can use in their daily activities because our population is growing older and technology continues to advance. The process will require nursing leaders and educational programs to work together with emerging developments while preserving the fundamental human nature that defines community care (15). The role of artificial intelligence (AI) and digital technologies in the management of nursing is growing, and this has created an opportunity for streamlining and predictive technologies, but at the same time, there are contesting issues such as resistance from staff, digital literacy, ethics, and legal outlines and training (36, 37, 114). Arab et al. (36) observe that AI tools are being used in enhancement of nursing education, practice and manpower management given by the integrative review. But many nurse are still show concerns about AI generated content and destruction of patient centered care. Katebi et al. (114) similarly establish the finding that AI although enhance nursing management but still produces loopholes relevant to ethical, regulatory and governance issues that remain unaddressed throughout the literature.

Telehealth and remote monitoring systems will become the main driving force that creates this upcoming future. The impact of innovative tools varies depending on their application, ranging from improved monitoring to enhanced patient engagement (20, 27). In order to make the nurse ready to accept this fact, the nurse must be made digitally health literate so that the nurse is not at any greater loss than when dealing with a patient substantially in front of them (36, 56). The need to improve interdisciplinary partnerships has become mandatory for all organizations. The healthcare model, which integrates nurses, doctors, and social workers, will deliver better patient results through its enhanced ability to coordinate operations (14, 52). The new generation in home care has to incorporate this into their teamwork, and therefore, shared decision-making will become the norm. On the one hand, structured training can help in reducing hospital readmissions successfully, as preliminary indications show (10). Karam et al. (52) showed through participatory action research that interdisciplinary collaboration between clinical practitioners and primary care nurses can lead to better care to the patient but require strict and organized facilitation and management that challenge the concept that teamwork required proximity and willingness only. Melnyk et al. (56) established that EBP capabilities are roots to this collaborative willingness, but still, this becomes inspiration rather than to implement as standard in social settings.

The social determinants of health (SDOH) are another issue we have to face. Nurses have a unique role that enables them to identify vulnerable housing situations and food insecurity problems and create solutions for these issues through their patient care plans (33). The use of standardized SDOH measurements and training of nurses who would advocate in a community will be crucial toward the accomplishment of health equity (28). There are large gaps in research, and while telehealth and AI are still in the “promise phase,” there is a need for long-term research on the effects on expenditures, the workload on the nurse, and, most importantly, the patient–nurse relationship, as well as the development of nursing protocols, especially in the home versus the hospital (37, 41, 114). Patient education and confined help must be incorporated to enhance self-management in future studies while performing experiments (12). Che and Cheung (33) establish findings through a long systematic review that social aspects consistently emerge as robust predictors of home care service facilitation than clinical need alone. These facts directly demand to reconstruct the system to check how services are offered and developed. Wei et al. (37) further conclude that the how use of AI is affecting the relationship of nurses and patients, which is important and still not fully explored and discussed thoroughly in literature. The management field benefits from predictive modeling and data analysis as essential tools for operational development. The algorithms enable managers to forecast patient requirements while they can simultaneously manage staff assignments throughout the day (20, 36, 114). Simulation and virtual reality, on the other hand, have emerged as the next big training tool for nursing staff in complex home-based scenarios (36, 66). High-quality mentoring programs demonstrate exceptional results by improving nurse retention rates while building a workforce that maintains stability and strength (53, 66). The evolution to patient-centered management occurs when care plans are designed between patients and families, establishes trust and enhances adherence (59). This can be leveled out and become more refined through remote devices (58). Fosah and Llahana (53) establish facts through a systematic review that lack of leadership in advanced nursing practices that includes unclear roles, organizational resistance, and lack of sufficient support by mentors is an issue that remains thoroughly underexplored, pointing a critical gap between the leadership role that future home-based care systems will need and what current organizational structures are actually providing.

Community-based care will rely on impartiality and be technologically focused in the near future. The essential priorities that need attention include telehealth expansion, interdisciplinary team development, assessment of social health determinants, and implementation of data-based methods. The entire research program needs extended funding because its objectives require support for academic work, professional development, and effective public policy research (53, 114). The community-based care system creates significant improvements for patients through the establishment of an analytical and empathetic organizational culture. Katebi et al. (114) and Fosah and Llahana (53) reach to a common conclusion: that the technical and institutional infrastructure for future home-based care is improving more rapidly than the human, ethical, and mentorship frameworks needed to govern it. Fulfilling this gap must become a shared priority for researchers, educationists, and policymakers in the coming years.

## 12 Conclusion

This review highlights the evolving role of home care in modern healthcare systems. Nurses utilize their clinical knowledge to develop patient educational programs. The narrative review as a whole suggests three interconnected domains: that nursing, management, and digital health really do not operate independently in home-based care but work in a collaboration that the populations most reliant on home-based care face complex vulnerabilities that cannot be handled by single-domain adequately, and the pace of technological change is stripping our ability to govern, reason, and create workforce frameworks.

Home care has developed into its present form through the dominant presence of digital innovation. Technology should support, not replace, patient–provider interaction. We need to establish methods that allow all individuals to benefit from technological progress. However, unequal access to digital resources remains a major barrier. Future models should integrate social, environmental, and digital health perspectives. The primary limitation of home care is that it leads to less personal interaction and emotional support from medical staff. The system offers both medical assistance and daily living skills to support people in their daily tasks. An effective team-based approach across disciplines is essential. Through their collaboration, they can develop solutions that will simplify life and build a healthcare system that operates through virtual means while being sustainable, equitable, and maintaining market competition. Certain gaps still exist in literature that need to be addressed by future research. Firstly, the long-term cost-effectiveness of digital health is less evidence-based. Secondly, how AI and informatics affect the nurse–patient relationship, particularly remotely, is rarely discussed thoroughly. Equal access to home-based services, especially in the low-income setting, rural areas, and diverse cultural populations, is still not properly achieved. Furthermore, leadership and management need to sustain and implement technology on a large scale that is still insufficient. Robust evidence shows that supporting technology can provide better home care. Sustainable multidisciplinary research funding, policy amendments that keep up with clinical discoveries, and an effort to keep the home-based care system that is not only effective but easily accessible to all communities are the gaps that require research and urgent attention.
