# Correction: Digital transformation of home-based care: a narrative review of nursing, technology, and management synergies

**DOI:** 10.3389/fpubh.2026.1947312

**Published:** 2026-09-03

**Authors:** Fangfei Wang, Ronghui Zhu, Xin Sui, Jianying Jiang, Tingting Zhang, Zhenzhen Xu, Gohar Iqbal

**Affiliations:** 1 Shenzhen Nanshan Medical Group Headquarters, Shenzhen, Guangdong, China; 2 College of Social Sciences, Shenzhen University, Shenzhen, Guangdong, China; 3 Clinical Medical Academy, Changchun Medical College, Changchun, China; 4 Academy of Nursing, Changchun Medical College, Changchun, China; 5 Shenzhen Nanshan People's Hospital, Shenzhen, China; 6 University of Veterinary and Animal Sciences, Lahore, Pakistan

**Keywords:** data-driven, digital transformation, digitally empowered nursing leadership, digitally proficient, interdisciplinary collaboration, technology

Fangfei Wang and Ronghui Zhu should be affiliated with “Shenzhen Nanshan Medical Group Headquarters, Shenzhen, Guangdong, China” not with “Shenzhen University, Shenzhen, China”.

Xin Sui should be affiliated with “College of Social Sciences, Shenzhen University, Shenzhen, Guangdong, China” not with “Shenzhen Research Institute, Guangdong Ocean University, Shenzhen, China”.

The Academy departmental units were missing in the Changchun Medical College affiliation for Jianying Jiang and Tingting Zhang. These have now been added.

The original version of this article has been updated.

